# 

**Published:** 1909-10-15

**Authors:** 


### ANNOUNCEMENTS.

THE MISSOURI DENTAL SOCIETY has elected the following officers: R. E. Darby, Springfield, president; O. J. Fruth, St. Louis, first vice president; C. C. Allen, Kansas City, second vice president; J. F. Wallace, Canton, recording secretary; J. D. Patterson, Kansas City, corresponding secretary, and J. T. Fry of Moberly, treasurer.

THE ILLINOIS DENTAL SOCIETY has elected the following officers: President, E. H. Allen, Freeport; vice president, C. C. Corbitt, Edwardsville; secretary, J. F. Walsh, Decatur.

THE LAKE ERIE DENTAL ASSOCIATION is now officered as follows: President, Dr. C. L. Mead, Union City; vice president, Dr. Ross Porter, Oil City; secretary, Dr. V. H. McAlpin, Warren, Pa.; treasurer, Dr. D. C. Dunn, Meadville.

ILLINOIS BOARD OF EXAMINERS. The annual meeting of the Illinois State Board of Dental Examiners for the examination of applicants for a license to practice dentistry in the state of Illinois will be held in Chicago at the Dental Department of the University of Illinois, corner Honore and Harrison Sts., beignning Monday, November 8, 1909, at 9 A. M.

The following preliminary qualifications shall be required of candidates to entitle them to examination by this board for a license to practice dentistry in the state of Illinois. Graduates of a reputable dental or medical school or college, or dental department of a reputable university, who enter the school or college as freshmen on or after the school year of 1906, 07, must have a minimum preliminary education of not less than graduation from an accredited high school or a certificate from the state superintendent of public instruction, equivalent officer or deputy, acting within his proper or legal jurisdiction, showing that the applicant had an education equal to that obtained in an accredited high school; which certificate shall be accepted in lieu of a high school diploma.

Graduates will be furnished with proper blanks and such other information as is necessary on application to the secretary. All applications must be filed with the secretary five days prior to date of examination. The examination fee is twenty dollars, with an additional fee of five dollars for a license.

Address all communications to

T. A. Broadbent, *Sec'y*,
705 Venetian Bldg.,
Chicago, Ills.

—✦—

Northern Ohio Dental Society. The following officers were elected at the last meeting at Cleveland: President, W. A. Siddall; vice president, W. G. Ebersole; secretary, J. W. McDill; treasurer, S. B. Dewey.

—✦—

Ohio State Dental Board. The Ohio State Dental Board will hold its regular fall meeting in Columbus, on October 19 to 22, 1909, for the examination of applicants for license. All applications with the fee of $25.00 should be in the hands of the secretary not later than October 9th.

For further information and blank applications address

F. R. Chapman, *Sec'y*,
305 Schultz Bldg.,
Columbus, Ohio.

---

New York Odontological Society. At the annual meeting of the New York Odontological Society, held April 20, 1909, the following officers were elected for 1909-10: W. D. Tracey, president; W. B. Dills, vice president; W. B. Dunning, recording secretary; J. G. Fulton, corresponding secretary; F. C. Walker, treasurer; W. W. Walker, curator, and F. C. Kemple, editor. Executive Committee—B. C. Nash, chairman, S. G. Perry, and A. L. Swift. Clinic Committee—J. W. Taylor, chairman, H. W. Gillett, and C. F. Ash.

F. C. Kemple, *Editor*.

---

Ohio State Dental Society. The forty-fourth annual meeting of the Ohio State Dental Society will convene in the assembly rooms of the Great Southern Hotel, Columbus,on December 7, 8 and 9, 1909. The program of papers and clinics will be second to none of those of the past.

A more extended notice will appear in the November number of the Register.

Mark these dates off your appointment book *now* and come to stay through the entire meeting.

F. R. Chapman, *Sec'y*,
305 Schultz Bldg.,
Columbus, Ohio.

